# Identification of Alkaline Salt Tolerance Genes in *Brassica napus* L. by Transcriptome Analysis

**DOI:** 10.3390/genes13081493

**Published:** 2022-08-21

**Authors:** Yu Xu, Shunxian Tao, Yunlin Zhu, Qi Zhang, Ping Li, Han Wang, Yan Zhang, Aldiyar Bakirov, Hanming Cao, Mengfan Qin, Kai Wang, Yiji Shi, Xiang Liu, Lin Zheng, Aixia Xu, Zhen Huang

**Affiliations:** 1 State Key Laboratory of Crop Stress Biology for Arid Areas/College of Agronomy, Northwest A&F University, Xianyang 712100, China; 2 Forestry Bureau of Xingtai City, Xingtai 054000, China

**Keywords:** *Brassica napus*, alkaline salt, RNA-seq, carbohydrate metabolism, photosynthesis, ROS

## Abstract

Soil salt alkalization is one major abiotic factor reducing the productivity of crops, including rapeseed, an indispensable oil crop and vegetable. The mechanism studies of alkali salt tolerance can help breed highly resistant varieties. In the current study, rapeseed (*B. napus*) line 2205 exhibited more tolerance to alkaline salt than line 1423 did. In line 2205, the lesser plasma membrane damage index, the accumulated osmotic solute, and higher antioxidant enzyme activities contributed to alkaline tolerance. A more integrated mesophyll-cell structure was revealed under alkali salt stress by ultrastructure observation in line 2205, which also implied a lesser injury. Transcriptome analysis showed that more genes responded to alkaline salt in line 2205. The expression of specific-response genes in line 1423 was lower than in line 2205. However, most of the specific-response genes in line 2205 had higher expression, which was mainly enriched in carbohydrate metabolism, photosynthetic processes, ROS regulating, and response to salt stress. It can be seen that the tolerance to alkaline salt is attributed to the high expression of some genes in these pathways. Based on these, twelve cross-differentially expressed genes were proposed as candidates. They provide clues for further analysis of the resistance mechanism of rapeseed.

## 1. Introduction

Salinity is among the most detrimental stresses in plants, as salt hampers plant growth, development, yield, and quality [1]. Soil salinization is intensifying because of unsuitable irrigation practices and other causes of climate change and environmental pollution [2]. The Global Map of Salt-affected Soils displays that 85% of topsoils (0–30 cm) and 62% of subsoils (30–100 cm) are salines, 34% and 19% of which are sodic and saline, respectively. Salt-affected soils are distributed in over 100 countries, especially in the arid and semi-arid regions, including China. However, the basic national conditions of China’s large population make it difficult to grow crops in non-saline land. Therefore, rational exploitation and improvement of the salinized land can relieve the pressure on cultivated land resources. According to reports, the total area of available saline-alkali soil is 3.67 × 107 ha in China, 30% of which shows great agricultural potential [3,4].

Salt-affected soils are mainly categorized into two groups: saline and sodic (alkaline). Generally, there is the presence of excess sodium on exchange sites in both soils, while the anions are different. The dominant anions are chloride and sulfate in saline soils. However, a high concentration of carbonate/bicarbonate anions in sodic soils causes high pH, a high sodium absorption ratio, and low electrical conductivities compared with those in saline soils [5]. In salt-stress plants, the imbalance of osmotic potential induces water stress, a high concentration of Na^+^ causes severe ion toxicity, and the interaction between salts and mineral nutrients results in nutrient imbalance and membrane injury [1,6]. In addition, plants growing in alkaline salt often suffer more severe damage due to high pH [7]. The disruption of pH homeostasis affects the absorption of inorganic ions and causes the precipitate of metal ions, resulting in a decrease in the availability of plant nutrients [8]. Thus, plants subjected to alkali salt stress not only have physiological drought and ion toxicity but also have to maintain intracellular pH balance.

Many studies have revealed the responding mechanisms to salt stress (NaCl) temporally and spatially in the model plant *Arabidopsis thaliana*. Briefly, a series of early responses (including K^+^ transport, Ca^2+^ signaling, H^+^ transport, phospholipid modifications, reactive oxygen species (ROS) production, and protein kinase activity) are induced after the initial perception of the sodium signal [1,9]; downstream of these signals, biosynthesis and transport of phytohormone are altered, and some gene expressions are changed in phytohormone-dependent and -independent manners [10]. Finally, these cascade signals lead to adaptive responses to salt stress, such as production and transport of ions and adjustment of growth and development [11,12]. Under salt stress, the excessive ROS disrupts redox homeostasis and causes oxidative damage to plant cells [13]. To alleviate the negative effect of oxidative stress, non-enzymatic (like glutathione, ascorbic acid, α-tocopherol, and flavonoids) and enzymatic defense systems (e.g., superoxide dismutase (SOD), peroxidase (POD), and catalase) perform the functions in scavenging or detoxifying the overgenerated ROS. In addition to these reactions, osmotic unbalance caused by salt stress leads to stoma close, which reduces the amount of CO_2_ available for photosynthesis, and CO_2_-fixing-enzyme activity decreases. Moreover, Na^+^ disrupts the proton-motive force and chloroplast function, which decreases the photosynthetic rate [14]. Hence, plants change the distribution of carbon to cope with the weakened photosynthate and sustain basic growth. In *Arabidopsis*, salt stress induces carbon partitioning to sugars instead of starch [15]. van Zelm et al. revealed that spatial carbon influx might be related to the tissue-specific sodium content [1].

Relative to the mechanisms responding to salt, limited information is known on the impact of alkaline salt. Most studies about alkali stress are conducted on the genome, physiological, and biochemical levels. For example, alkali stress causes the loss of wheat seeding biomass and accumulated inorganic ions with different trends compared with salt stress [16]. The different effects on the growth and photosynthesis comparing alkali and salt stress have also been investigated by Guo et al. [17]. The investigation of two Chinese cabbage cultivars found that photosynthesis was affected under alkaline stress and the varying photosynthetic capacity was associated with the synthesis of organic acids and carbohydrates [7]. The physiological responses to NaCl and NaHCO_3_ were also studied in tomatoes, which showed that organic acids and regulation of ROS might be related to the alkali stress tolerance of tomatoes [18]. Many quantitative trait loci and genome-wide association studies related to alkali salt have been reported in rice [19,20], and some alkali salt-regulation genes have also been cloned in soybean [21,22,23], rice [24,25], and alfalfa [26,27].

*B. napus Rapeseed* (*Brassica napus* L.) is the second oil crop, an important edible vegetable, and a crucial source of biofuel [28]. Oilseed in China mainly relies on imports because of the low production from lower plantings. Expanding the cultivated area of rapeseed by planting salt- and alkali-tolerant rapeseed in salt–alkali soils, especially in the idle lands in the northern and coastal areas of China, can remit the over-reliance on the international market. The expound on the alkaline salt tolerance mechanism is beneficial to the breeding of new resistant varieties. Although many rapeseed cultivars with high diversity in salt resistance have been reported [29], the details about genes and mechanisms of tolerance to alkalinity are largely unknown in rapeseed. Transcriptomic analysis can provide mRNA expression of genes at high throughput and is widely applied to screen candidate genes involved in stress responses [29,30]. In the present study, an RNA-seq was performed on alkaline-tolerant and -sensitive rapeseed cultivars to screen alkaline-salt-tolerance-related genes, which will lay a solid foundation for the breeding and mechanism of alkaline salt tolerance in *B. napus*.

## 2. Materials and Methods

### 2.1. Plant Materials and Growth Conditions

The salt-tolerant line 2205 (T) and salt-sensitive line 1423 (S) were grown in a greenhouse at 25 ℃ and 60% relative humidity under a 16 h light/8 h dark photoperiod. Firstly, the seeds were germinated on filter papers with distilled water for three days until the cotyledons unfolded. Secondly, the buds were transplanted into the matrix (Mineral/vermiculite/Perlite = 3:1:1). When the seedlings reached the two-leaf stage after ten days, the uniform seedings were placed to the Hoagland nutrient solution (pH 6.56) [31] for hydroponics. After growth for 6 days, alkaline salt stress was performed in the Hoagland nutrient solution containing 75 mmol/L NaHCO_3_ (pH 8.12), while the control group contained only the nutrient solution. After treatment with 75 mmol/L NaHCO_3_ for 0, 12, and 24 h, the young leaves of line 2205 (T0, T12, and T24) and 1423 (S0, S12, and S24) were collected for RNA-seq. The leaves from 10 plants were pooled to form one biological replicate and three replicates were generated for each treatment. In total, 18 samples were collected and stored at −80 °C as independent biological samples.

### 2.2. Measurement of Physiological Indexes

To further evaluate and analyze resistance differences to alkali stress between lines 2205 and 1423, six conventional physiological indicators related to stress response, including relative electric conductivity (REC), soluble sugar (SS), soluble protein (SP), proline (Pro), superoxide dismutase (SOD), and peroxidase (POD), were examined using the young leaves after being treated for 0, 12, and 24 h. REC was detected using the method described by Lutts et al. [32], SS and Pro were measured by the method of Zhao et al. [33], the examination of SP was performed by the description of Woolhouse et al. [34], SOD was determined by the method of Beauchamp and Fridovich [35], and POD was detected based on the procedure provided by Zhao et al. [36]. These experiments were repeated three times with three biological replicates. For each biological replicate, three plants were collected.

### 2.3. Electron Microscopic Observation of Seedling Cells under NaHCO_3_ Stress

At the three-leaf stage, the third true leaf was collected at 0 h (CK) and 7 d after alkaline salt stress and washed with distilled water. Then, the tissue block with a width of 1 mm and a length of 5 mm along the middle of the main leaf vein was cut. The same part of each leaf was taken once, and the process was repeated using three plants. The cut tissue was quickly placed into a 4% glutaraldehyde solution (pH = 6.8) for 12 h after vacuuming. Then, the tissue was washed with a 0.1 mM phosphate buffer saline (PBS) buffer (pH = 6.8) for 10 min four times. The leaves were transferred to 0.1 mM osmium acid for 1.5–2.0 h, washed with a 0.1 mM PBS buffer (pH = 6.8) for 10 min five times, and then dehydrated in ethanol solutions with gradient concentrations (30%, 50%, 70%, 80%, and 90%) for 15 min. The samples were then treated with 100% ethanol for 30 min and 100% acetone for 30 min twice, infiltrated with acetone, permeated with pure glue for 24 h, and then embedded with resin. The half-thin section was sliced by a microtome, stained by uranyl acetate and lead citrate, and observed by a transmission electron microscope (HT7700, Hitachi, Tokyo, Japan).

### 2.4. RNA Extraction, Library Construction, and Sequencing

The total RNA of these samples was extracted with an RNAprep Pure Plant Kit (TIANGEN, DP441) and subjected to quality control with a Bioanalyzer 2100 system (Agilent Technologies, Palo Alto, CA, USA). The RNA concentration was measured by NanoDrop 2000 (Thermo). A total amount of 1 μg of RNA per sample was used as the input material for RNA sample preparations. The libraries were sequenced on an Illumina HiSeq X Ten platform at Beijing BMK Biotechnology Co., Ltd. (Beijing, China).

### 2.5. RNA-Seq Analysis

Quality control was performed by removing reads containing an adapter, ploy-N, and low quality from raw data. The clean paired-end reads were mapped to the designated reference genome of *B. napus* (http://www.genoscope.cns.fr/brassicanapus/data/, accessed on 20 September 2021) by the Hisat2 tools soft [37]. Only reads with a perfect match or one mismatch were further analyzed and annotated based on the reference genome. Gene expression levels were estimated by fragments per kilobase of transcript per million fragments mapped (FPKM) [38]. Pairwise differential expression analyses were performed using the DEseq [39]. Comparison schemes for these two lines, the salt-tolerant line 2205 (T) and salt-sensitive line 1423 (S), at three-time points (0, 12, and 24 h) were set up to analyze the gene expression, including S0 vs. T0, S12 vs. T12, S24 vs. T24, S0 vs. S12, S0 vs. S24, T0 vs. T12, and T0 vs. T24. The resulting *p*-values were adjusted using the method by Benjamini and Hochberg for controlling the false discovery rate (FDR). Genes with an FDR < 0.01 and |log_2_ fold change| ≥ 1 were considered to be differentially expressed.

Further analyses of DEGs were performed using the tools on the platform BMKCloud (www.biocloud.net, accessed on 27 February 2022). Venn and clustering heatmap plots were generated by R packages “VennDiagram” and “heatmap”. The box plots of DEG sets were drawn using GraphPad Prism v9.0 software (San Diego, CA, USA).

### 2.6. Functional Enrichment Analysis of DEGs

Gene Ontology (GO) enrichment analysis of DEGs was implemented by the GOseq R packages with the significant threshold of *p* < 0.05 [40], and the significantly enriched terms were graphed using GraphPad Prism v9.0 software. In addition, Kyoto Encyclopedia of Genes and Genomes (KEGG) pathways were enriched using the KOBAS online analysis database [41], and the top 20 pathways were displayed.

### 2.7. Verified DEGs by qRT-PCR

To verify the differential expression detected by the RNA-seq, the remaining RNAs of the 18 samples were reverse-transcribed in a 20 μL reaction mixture with a FastKing RT Kit (With gDNase, TIANGEN, KR116) following the manufacturer’s instructions. The transcriptional expression of six randomly selected DEGs was explored by quantitative real-time PCR (qRT-PCR). The *Actin* gene was used as the internal control for qRT–PCR data analysis. The primes for these genes are listed in Table S1. The reaction was performed in 8-tube strips on a QuantStudio™ 7 Flex (Thermo Fisher Scientific, Waltham, MA, USA) using SYBR Green Master ROX (TaKaRa, Dalian, China) in accordance with the manufacturer’s instructions. Three technical replicates for each biological-replicate sample were analyzed. Relative fold differences of the selected DEGs among two lines or experiments were calculated using the 2^−ΔΔCt^ method [42]. Pearson’s correlation coefficient was used to express the correlation of log_2_(fold change) between samples. The results were shown by GraphPad Prism 9.0.

### 2.8. Statistical Analysis

Data processing was conducted using Microsoft Excel 2019, and figures were generated using GraphPad Prism 9. All triplicate data were subjected to one-way analysis of variance (ANOVA) using IBM SPSS Statistics 21. The data are presented as the mean (±SD) of three replications. Means were separated by the least significant difference test at 5% level of significance.

## 3. Results

### 3.1. Differences of Alkaline Salt Tolerance and Physiological Indicators between Lines 2205 and 1423 under Alkaline Salt Stress

**Figure 1:**
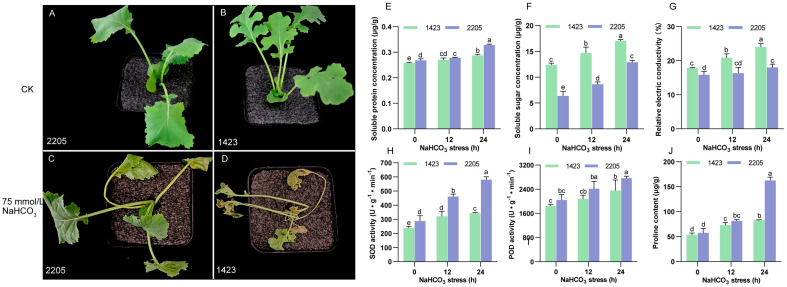
Phenotypical photographs and physiological responses of lines 2205 and 1423 under control and alkaline salt stress. Line 2205 (**A**) and line 1423 (**B**) seedlings in the Hoagland nutrient solution, 7-day-stress 2205 (**C**) and 1423 (**D**) seedlings using 75 mmol/L NaHCO_3_. Bar graph represents the content of soluble protein (**E**), soluble sugar (**F**), relative electric conductivity (**G**), the activity of superoxide dismutase (**H**), and peroxidase (**I**), and proline content (**J**). The assays were repeated three times with three biological replicates. Different letters indicate significant differences detected by the least significant difference test (*p* < 0.05).

The growth of lines 2205 and 1423 showed no difference under normal conditions (Figure 1A,B). However, they showed different phenotypes after 7 days under alkaline salt stress. At this stage, the leaves of line 1423 almost withered, while the leaves of line 2205 just began to yellow and wilt (Figure 1C,D), indicating that line 2205 was more tolerant to alkaline salts than line 1423.

To further evaluate the difference in alkaline salt tolerance between these two cultivars, the physiological indexes related to stress response were measured. The results showed NaHCO_3_ stress increased relative electric conductivity (REC), the content of soluble sugars (SS), soluble protein (SP), proline (Pro), and the activity of POD and SOD in both cultivars, and the tendency increased over the stress time (Figure 1). The content of SP was higher in line 2205 than in line 1423, and the gap was increased after 24 h of stress (Figure 1E). Under normal and alkaline salt conditions, the concentration of SS and REC was significantly lower in line 2205 compared with that in line 1423, which was opposite to SOD activity (Figure 1F–H). There was no significant difference in Pro content and POD activity between line 2205 and line 1423 under normal growth conditions and 12 h of stress, which were significantly elevated in line 2205 after 24 h of stress compared with that in line 1423 (Figure 1I–J). These results implied that the physiological responses to alkaline salt were different between line 2205 and line 1423.

### 3.2. Ultrastructural-Feature Differences between Line 2205 and Line 1423 under Alkaline Salt Stress

**Figure 2:**
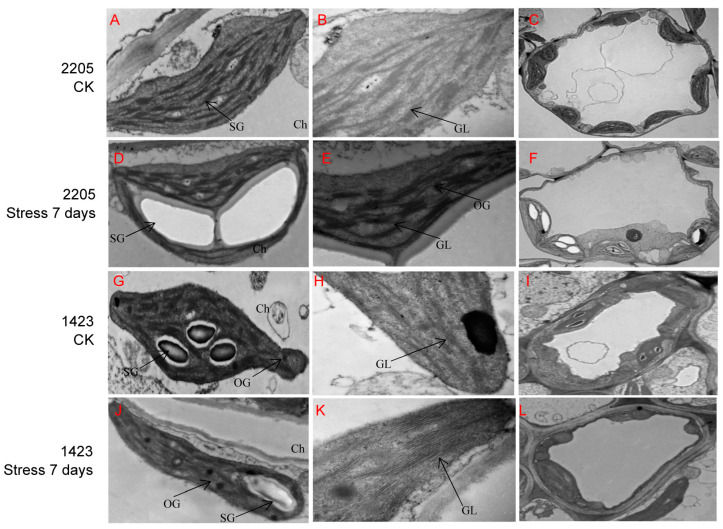
Ultrastructural observation of the tolerant line 2205 and sensitive line 1423 under control and NaHCO_3_ stress. The ultrastructure of leaves of line 2205 (**A**–**C**) and line 1423 (**G**–**I**) without alkaline salt stress, 7 d NaHCO_3_-treated 2205 (**D**–**F**) and 1423 (**J**–**L**). Ch, chloroplast; GL, basal grains; OG, osmophilic granules; SG, starch granules.

Observation of microstructures showed that the mesophyll cells of lines 2205 and 1423 exhibited typical features under normal conditions. The chloroplasts were spindle-shaped structures, the basal particles were arranged in an orderly manner, the thylakoids were arranged compactly, and starch grains were present in the chloroplast of both lines (Figure 2A,B,G,H). However, the cells of line 2205 were approximatively round, and more and tighter chloroplasts were found, which showed a long oval shape in line 1423 (Figure 2C,I). After alkaline salt stress for 7 days, line 1423 suffered a greater disruption, obvious plasmolysis occurred, and the organelles in the cells decreased or dissolved. However, many intact organelle structures could still be observed in line 2205 (Figure 2F,L). In contrast to the shrunken manifestation occurring on the thylakoid and starch grains of chloroplasts in the salt-sensitive line 1423, the thylakoid of line 2205 swelled, and the starch grains in the chloroplasts became larger and more transparent. Moreover, the basal particles of line 2205 still maintained the regular arrangement to some extent, while those of line 1423 were arranged loosely and distorted. In addition, line 2205 had fewer osmiophilic granules compared with line 1423 (Figure 2D,E,J,K). From these results, we speculated the stronger resistance of line 2205 was related to the more complete chloroplast structure under NaHCO_3_ stress.

### 3.3. Illumina Sequencing and Differentially Expressed Genes between Line 2205 and Line 1423

In order to explore the discrepant genes responding to alkaline salt between line 2205 and line 1423, RNA-seq was performed after 0 h (control), 12 h, and 24 h NaHCO_3_ stresses on three independent biological replicates. In total, 116.40 GB of clean data from 18 RNA libraries were obtained after filtering. The clean data of each sample reached 4.90 GB, the GC content was between 45.85–46.88%, and the percentage of Q30 base was more than 91.71%. The efficiency of sequence comparison between the clean reads of each sample and the reference genome ranged from 83.09% to 88.39% (Table S2).

**Figure 3:**
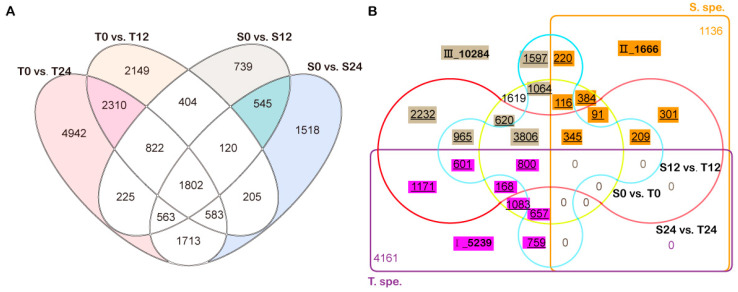
Venn diagram of DEGs among different groups. (**A**) Numbers of DEGs among the comparisons of control and 12 h or 24 h NaHCO_3_ stress. The colored parts represent the genes that responded specifically to salt in line 2205 (T. spe.) and line 1423 (S. spe.). (**B**) The intersection of DEGs among T. spe. (left padding part in A, purple rectangle in B), S. spe. (right padding part in A, orange rectangle in B), S0 versus T0 (yellow circle), S12 versus T12 (blue four-sided arc), S24 versus T24 (red dumbbell arc). The purple, orange, and gray filling DEGs with underlines mark the concrete genes in subsets I, II, and III for further analysis, respectively.

To identify changes in transcriptional expression under alkali stress, the differentially expressed genes (DEGs) were identified by comparing the expression of genes between 12 and 24 h and those at 0 h in both cultivars. In the sensitive line S, 5220 (S0 vs. S12) and 7049 (S0 vs. S24) genes were altered at the transcriptional level, whereas 8395 (T0 vs. T12) and 12960 (T0 vs. T24) were differentially expressed in the tolerant line T (Figure 3A and Table S3). It was found that line T had nearly twice as many DEGs as line S. Among these DEGs, 9401 and 2802 genes specifically respond for alkaline salt in line T (T. spe.) and line S (S. spe.), respectively (Figure 3A). Futher, 5239 DEGs had significantly different expression between T and S in T. spe, as subset I. Similarly, 1666 DEGs in S. spe differently expressed between T and S, as subset II (Figure 3B). Additionally, 10,284 DEGs responded simultaneously to alkaline salt in the two lines, and their expression exhibited significant differences. These genes were defined as subset III (Figure 3B).

### 3.4. Co-expression Clustering of Filtered DEGs

**Figure 4:**
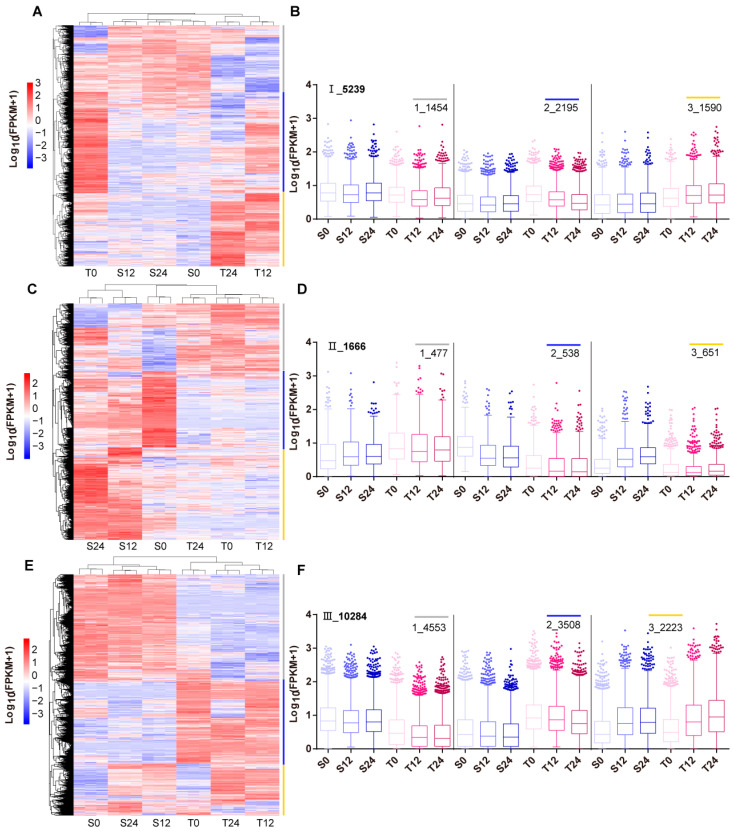
Expression clustering of selected DEGs. Clustering heatmaps of Log_10_ (FPKM + 1) values for these DEGs in subsets I (**A**), II (**C**), and III (**E**), respectively. (**B**,**D**,**F**) Box plots display the distributions of expression for the associated cluster in A, C, E. According to the heatmap, each subset can be clustered into three groups. The clusters 1–3 under each subset are marked by gray, blue, and yellow color lines. The numbers are shown on the top. In the box plots, the line marks the median, the top and bottom of the box represent the interquartile range, and the whiskers extend to 1.5 times the interquartile range.

To excavate the potential alkali-salt-regulating genes, the co-expression clustering analyses of filtered DEGs (subsets I, II, and III) were performed, and the values of Log_10_ (FPKM + 1) were used for clustering. Each subset could be divided into three clusters (Figure 4). On subset I, 72.25% of DEGs (clusters 2 and 3) had higher expression in T0, T12, and T24 (Figure 4A,B). However, the higher expressed DEGs accounted for just 28.36% in subset II, 73.37% of which (clusters 2 and 3) was expressed at a lower level in the tolerant line T (Figure 4C,D). On subset III, the top two clusters were downregulated (cluster 1) and upregulated (cluster 2) in S0, 12, 24 vs. T0, 12, 24 (Figure 4E,F). Taken together, the 3785 DEGs in subset I and the 3508 DEGs in subset III with an upregulated expression in line T might be responsible for the salt tolerance, and the other 1189 (in subset II) and 4553 (in subset III) genes with an upregulated expression in line S might have negative effects on salt resistance.

To analyze the functional differences of the DEGs in these two groups, the synchronously up- and downregulated genes (FDR ≤ 0.01, |log_2_ (fold change)| ≥ 1) in groups S0 vs. T0, S12 vs. T12 and S24 vs. T24 were further investigated. As a result, 2236 of 7293 (up-R. in T., higher expression in line T) and 2240 of 5742 DEGs (down-R. in T., lower expression in line T) were selected as the important DEGs (Table S4).

### 3.5. Gene Ontology Enrichment of the Important DEGs

**Figure 5:**
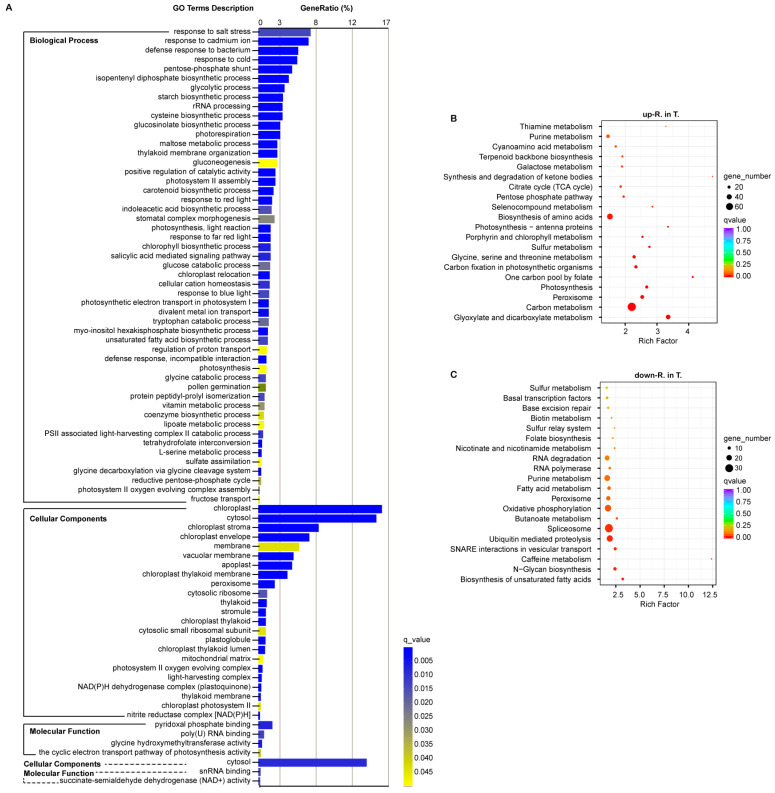
Significantly enriched terms and pathways for the selected DEGs. (**A**) Significant GO terms enriched by the up (full line) and down (broken line) expressed genes in line 2205. GO terms were classified into three groups (BP, CC, and MF). The top 20 KEGG pathways are shown for the genes from “up-R. in T” (**B**) and “down-R. in T” (**C**).

Functional cluster analyses of 2236 (up-R. in T.) and 2240 (down-R. in T.) DEGs were implemented using BMKCloud. For the 2236 DEGs, gene ontology (GO) analysis showed that 78 significant GO terms were enriched (*q*_value < 0.05), including 51 terms in biological process (BP), 23 terms in cellular component (CC), and 4 terms in molecular function (MF). Among these, the terms “response to salt stress” (6.91%), “chloroplast” (16.35%), “pyridoxal phosphate binding” (1.87%) accounted for the highest gene ratio in BP, CC, and MF, respectively. Moreover, these terms that referred to photosynthetic processes and carbohydrate metabolism were also highly enriched in BP, such as “photorespiration”, “photosystem II assembly”, “photosynthesis, light reaction”, “chlorophyll biosynthetic process”, “chloroplast relocation”, “photosynthetic electron transport in photosystem I”, “photosynthesis”, “PSII associated light-harvesting complex II catabolic process”, “photosystem II oxygen evolving complex assembly”; “glycolytic process”, “starch biosynthetic process”, “gluconeogenesis”, and “glucose catabolic process”. In addition to the cellular component related to the chloroplast, apoplast, mitochondria, and peroxisome were also significantly enriched. However, only three GO terms, “cytosol” in CC, “snRNA binding” and “succinate-semialdehyde dehydrogenase (NAD^+^) activity” in MF, were significantly enriched among the downregulated 2240 DEGs in line 2205 (Figure 5A).

Several studies have shown that the photosynthetic mechanisms of different salt-tolerant germplasms have different trends under salt stress [7,43]. Therefore, we hypothesized the higher expression of multiple genes participating in photosynthetic systems delayed the attenuation of photosynthesis in the tolerant line 2205. Chloroplasts are not only the site of photosynthesis, but also ROS production [44]. In addition, mitochondria and peroxisome significantly enriched in CC are also important sites for ROS releasing. Many researchers have indicated that ROS plays a dual role under abiotic stresses [13]. Thereby, we speculated that ROS homeostasis was stronger in line 2205 than line 1423, which increased the tolerance to alkaline salt.

### 3.6. Kyoto Encyclopedia of Genes and Genomes Enrichment of the Important DEGs

To further discern the functions of DEGs in line 2205 and line 1423, we conducted a Kyoto Encyclopedia of Genes and Genomes (KEGG) enrichment analysis. The top 20 enriched pathways for the 2236 (up-R. in T.) and 2240 (down-R. in T.) DEGs are shown in Figure 5. Similar to GO enrichment, the two-group DEGs were enriched in different pathways. The four most significantly enriched pathways were “glyoxylate and dicarboxylate metabolism”, “carbon metabolism”, “peroxisome”, and “photosynthesis” for the 2236 upregulated genes in line 2205 (Figure 5B). The other four pathways, “biosynthesis of unsaturated fatty acids”, “N-Glycan biosynthesis”, “caffeine metabolism”, and “SNARE interactions in vesicular transport”, were the top pathways significantly enriched for these 2240 upregulated genes in line 1423 (Figure 5C). To sum up, GO and KEGG analyses both showed that carbohydrate metabolism, photosynthetic processes, and ROS regulating were enriched for these 2236 DEGs, which were not enriched for those 2240 DEGs It is surmised that the higher expression of the genes in these pathways enhanced the tolerance to alkali salt stress of line 2205.

### 3.7. Candidate Gene Analysis

**Figure 6:**
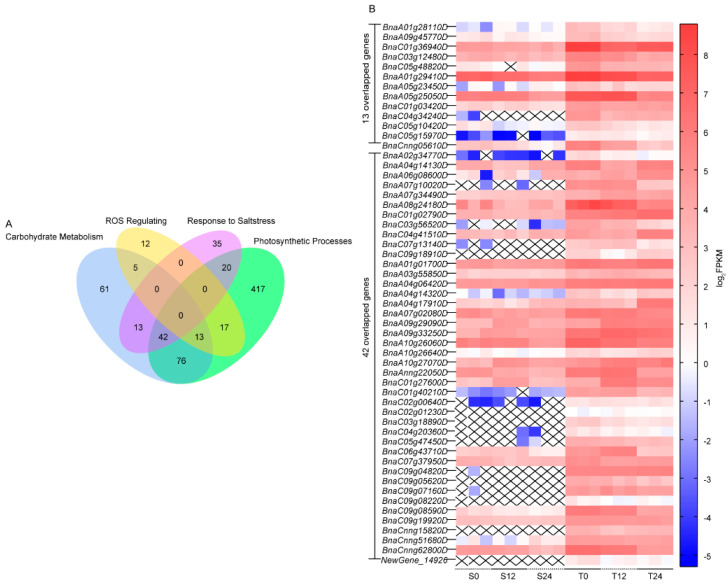
Candidate genes in the target pathways. (**A**) The Venn diagram shows the DEGs in the carbohydrate metabolism, photosynthetic processes, and ROS regulating under salt stress. (**B**) The heatmap displays the Log_2_FPKM of 13 DEGs (overlapped DEGs in photosynthetic processes and ROS regulating) and 42 DEGs (overlapped DEGs in carbohydrate metabolism and photosynthetic processes) for lines 2205 and 1423 under control, 12- and 24-hour stress. The “X” in the figure represents FPKM = 0.

Based on the functional enrichment results of the higher expression of DEGs in 2205, these DEGs in the top enriched pathways were further analyzed, including carbohydrate metabolism (210 DEGs), photosynthetic processes (585 DEGs), ROS regulating (47 DEGs), and response to salt stress (110 DEGs, Table S5). Salt response is a complex trait, which is affected by the coordinated gene networks in several metabolic pathways [1]. Hence, we focused on the intersectant genes among target pathways, including 13 overlapped DEGs in carbohydrate metabolism, photosynthetic processes, and ROS regulating; 42 overlapped DEGs among carbohydrate metabolism, photosynthetic processes, and response to salt stress (Figure 6A). The expressions of the 55 genes were higher in line 2205 than those in line 1423 (Figure 6B). The 55 genes were mainly annotated to carbon metabolism (ko01200), oxidative phosphorylation (ko00190), glyoxylate and dicarboxylate metabolism (ko00630), biosynthesis of amino acids (ko01230), carbon fixation in photosynthetic organisms (ko00710), 2-Oxocarboxylic acid metabolism (ko01210), glutathione metabolism (ko00480), and peroxisome (ko04146). Twelve genes (*BnaA06g08600D*, *BnaC05g47450D*, *BnaC01g40210D*, *BnaA01g01700D*, *BnaC01g02790D*, *BnaC01g36940D*, *BnaA05g25050D*, *BnaA01g29410D*, *BnaA07g10020D*, *BnaC07g13140D*, *BnaAnng22050D*, *BnaC06g43710D*) involved in at least two pathways were identified as the key candidate genes for the regulation of alkali salt tolerance (Table S5).

### 3.8. Validation of the RNA-Seq Data by qRT-PCR

**Figure 7:**
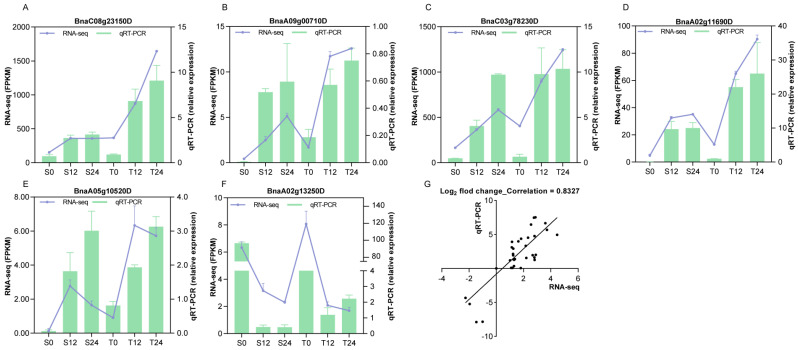
Validation of RNA-Seq transcriptional data using qRT-PCR. (**A**–**F**) Comparisons of RNA-seq data (By FPKM; green color bars) with qRT-PCR (By the relative expression of reference gene; purple lines) of randomly selected six genes. (**G**) The scatter plot shows the Pearson’s correlation of expression change by qRT-PCR (X-axis) and RNA-Seq (Y-axis).

To verify the differential expression detected by the Illumina RNA-Seq data, qRT-PCR was performed for six random genes using 18 RNA libraries. Similar expression trends were shown for the selected genes, and a high positive correlation (0.83) was obtained between RNA-seq and qRT-PCR data at different time points (Figure 7), suggesting that the RNA-Seq data were reliable.

## 4. Discussion

Salt stress is a severe hazard that adversely affects all stages of plant growth, such as inhibiting seed germination, slowing plant development, decreasing biomass yield, and decreasing crop yield [1,29]. Therefore, it is a great advantage to breed salt-tolerant rapeseed cultivars. Excavating the physiological and biochemical response mechanisms would be helpful to understanding the molecular tolerance traits and develop salt-tolerant genotypes. However, our present knowledge on the mechanisms regulating salt tolerance (especial alkaline salt stress) in rapeseed is still in its infancy. Hence, the selection and identification of high alkaline salt tolerance cultivars are crucial to expanding rapeseed production in saline-alkali soil.

### 4.1. Alkali Salt Tolerance of Line 2205 Is Reflected at Multiple Levels

Compared with line 1423, the 2205 cultivar exhibits strong tolerance not only to the neutral salt NaCl [45], but also to the alkaline salt NaHCO_3_. The discrepancy between lines 2205 and 1423 is reflected not only in the visual phenotype but also in physiological responses and ultramicrostructures of mesophyll cells. REC is an indicator used to quantify the cell membrane stability during adversary stress [46]. Reports have shown that stress (such as heat, drought, and salt) can damage the plasma membrane, inducing an increase in REC [46]. In the current study, REC was significantly higher in line 1423, leading to severe damage and weak selectivity of the membrane compared with those of line 2205 (Figure 1). Ultrastructural observation also discovered cell structures, and organelles of line 1423 appeared to dissolve when subjected to 7-day alkali salt stress (Figure 2), which may be correlated with the serious injury of the plasma membrane. Additionally, as important osmotic regulators, salinity-induced accumulation of SS and soluble protein SP have been reported [47,48]. In the current study, the increasing trend in SS and SP was detected after NaHCO_3_ stress, which is consistent with previous reports. However, the tolerant line 2205 presented a lower SS content but an opposite pattern of SP (Figure 1). Whether SP and SS play different roles in osmotic regulation in response to alkaline salt needs further investigation. Many studies have reported that Pro plays an important and positive role in plant growth as an osmoprotectant and antioxidative defense molecule under abiotic stress [49]. We also found it was accompanied by a higher proline content in the more tolerant line 2205 under alkaline salt (Figure 1). Salt stress triggers the excess generation of ROS, which causes oxidative damage to cells and even results in plant death. The antioxidant defense system constituted of non-enzymatic and enzymatic components saves plants from oxidative damage by detoxifying the excessive ROS and maintaining the balance of ROS [50]. Meanwhile, SOD and peroxidase POD are two important constituents of the antioxidant defense system [6]. In this study, higher POD and SOD activities in line 2205 endowed it with a higher scavenging ability for ROS (Figure 1). We speculated this might partially contribute to the tolerance to alkaline salt stress.

It was found that the integrity of mesophyll cells of line 2205 was better than that of line 1423 under NaHCO_3_ stress, and no plasmolysis was observed by microstructure observation (Figure 2). Previous studies found that the cell structure can be changed to minimize the damage when plants are subjected to saline-alkali stress [51]. The intact cell wall can be used as a barrier between the cell and the external environment to restrict ions from entering the mesophyll cells, thus protecting the cells from toxicity. The responses of mesophyll cells to saline-alkali stress in salt-tolerant and salt-sensitive materials are significantly different [52]. The mesophyll cells are important sites for photosynthesis; damage to mesophyll cells disturbs photosynthesis under stress [1]. In this study, the mesophyll-cell structure of line 2205 showed less damage compared with that in line 1423, indicating there may be more effective photosynthesis.

### 4.2. Synergistic Upregulation of Genes in Carbohydrates Metabolism, Photosynthetic Process, and ROS Equilibrium under NaHCO_3_ Stress

In this study, RNA-seq analysis identified more DEGs in the groups of T12 vs. T0 and T24 vs. T0 compared with the groups of S12 vs. S0 and S24 vs. S0, implying that more regulatory pathways had altered in line 2205 under 75 mM NaHCO_3_ stress (Figure 3A). This is consistent with the study of Mohamed et al. [29], which shows that the tolerant rapeseed YY9 has higher DEG regulation than the salt-sensitive one ZS11 on germination under salt stress. The higher number of DEGs in the tolerant line 2205 indicates that more pathways are involved in accommodating the alkali salt stress.

Further analysis discovered that 72.25% of the alkali-salt-specific response DEGs in line 2205 had higher expression than those in line 1423. However, 73.37% of line 1423-specific response DEGs showed lower expression in line 2205. Among these DEGs simultaneous responding to alkaline salt in both cultivars, 44.27% and 34.11% DEGs possessed higher expression in line 1423 and line 2205, respectively (Figure 3 and Figure 4). From the differentially up- and downregulated DEGs in these subsets, we speculated that their differences in alkali salt resistance were due to the diverse coordinative capabilities of positive and negative regulation of alkaline salt tolerance.

In this paper, in order to excavate the functions and pathways regulating alkaline salt in rapeseed, the functional enrichments were performed for the target-group DEGs in line 2205. A number of significant terms and pathways were annotated by GO and KEGG analysis. The genes with lower expression in line 2205 were mainly enriched in cytosol, spliceosome, and ubiquitin-mediated proteolysis (Figure 5). Numerous studies have revealed that the splicing of stress-responsive genes and spliceosome components can be altered by salt stress, which influences the salt tolerance of plants by adjusting the homeostasis of ROS and osmosis. For example, serine/arginine-rich (SR)-like protein, as a component of spliceosome, have been reported to negatively regulate salt stress [53,54]. Among the twelve serine/arginine-rich splicing factors among the selected DEGs in this study, nine genes (*BnaA03g00590D*, *BnaA06g21030D*, *BnaA07g06170D*, *BnaC02g27300D*, *BnaC07g07660D*, *BnaC08g16960D*, *BnaC08g38300D*, *BnaCnng19170D*, *Brassica_napus_newGene_614*) were from the set “down-R. in T.” (Table S6). It is speculated that the weak alkali salt tolerance of line 1423 is correlated with the high expression of these genes.

However, the screened DEGs from “up-R. in T.” were mainly enriched in carbohydrate metabolism, photosynthetic processes, ROS regulating, and response to salt stress (Figure 5). A number of studies confirmed that there are complicated mechanisms in regulating salt tolerance [1,45]. However, these extensive studies mainly focus on neutral salt NaCl, but not alkaline salt, especially for the oil crop, *B. napus*. Our analysis showed that the higher expression of some genes in carbohydrate metabolism, photosynthetic processes, and ROS adjustment improved the alkalinity tolerance of rapeseed. The results were different from the annotated pathways (fatty acid and amino acids metabolisms) in response to Na_2_CO_3_ in canola [55].

Considering the intricacy of quantitative traits, the intersection genes in target pathways were identified as candidate genes in regulating alkaline salt tolerance (Table S6). Malate dehydrogenase 2 (*MDH2*), a mitochondrial tricarboxylic acid cycle gene, has been proven the mRNA level of *MDH2* is higher in the tolerant rye than the sensitive one in response to aluminum stress [56], consistent with the candidate gene *BnMDH2* (*BnaA06g08600D*) in this study. Various studies have testified cytosolic glyceraldehyde-3-phosphate dehydrogenase (GAPC) is participated in plant response to stresses, including heat [57], clod [58], oxidative stress [59], and cadmium [60], but no to alkali salt stress. Two homologous genes, *BnGAPC1* (*BnaC05g47450D*) and *BnGAPC2* (*BnaC01g40210D*), were screened out in this study (Table S6). Both genes maintained high levels of expression in line 2205 even under the NaHCO_3_ treatment, but not in line 1423 (Figure 6). 1-Aminocyclopropane-1-carboxylate oxidase (ACO), which participates in the final step of ethylene synthesis, is induced by heat, cold stresses, wounding of leaves, soil-flooding, etc. in potato [61]. In our study, two *BnACO1* (*BnaA01g01700D* and *BnaC01g02790D*) showed high expressions in line 2205 (Figure 6). Plants will burst ROS under stress. Excessive production of ROS will lead to damage for plants. However, when these molecules are at the right levels, they play an active role in signal transduction [13]. Glycolate oxidase (GOX), a typical enzyme of peroxisomes, can trigger. In plants, pathogen-induced expression of *GOX* activates the expression of several defense genes and improves the resistance to pathogens by increasing H_2_O_2_ [62,63]. In our study, three *BnGOXs* (*BnaC01g36940D*, *BnaA05g25050D*, and *BnaA01g29410D*) with significantly higher expression in line 2205 were identified, which may result in more glucose digestion and less SS, and simultaneously boost the POD and SOD activities in line 2205 (Figure 1 and Figure 6). Glutamate:glyoxylate aminotransferase (GGAT) plays an important role in photorespiratory carbon cycles and amino acid metabolism. The growth is strongly affected in *aoat1-1* seedlings with a weak GGAT activity [64]. In our results, two *BnGGTA1* (*BnaA07g10020D* and *BnaC07g13140D*) showed no expression in line 1423, and the FPKM of two *BnGGTA2* (*BnaAnng22050D* and *BnaC06g43710D*) were lower in line 1423 compared with line 2205 (Figure 6).

At present, there are few studies on the salt-tolerance mechanism of rapeseed. Only a handful of neutral salt-regulated genes such as alternative oxidases (*BnaAOXs*) [65], calcineurin B-like proteins (*BnaCBL)*, and CBL-interacting protein kinases (*BnaCIPK*) [66] were cloned, and even fewer alkali-salt-tolerance genes have been reported in *B. napus*. Our study proposed firstly candidate genes for alkaline salt tolerance in *Brassica napus* by RNA-seq, including twelve target genes in metabolic pathways (such as carbohydrate metabolism, photosynthetic processes, and ROS regulating), which positively responded to alkaline stress in rapeseed. Our study provides new insight into the mechanism of alkali salt tolerance in *B. napus*. Cloning and functional analyses of these candidate genes have been arranged in follow-up projects to more accurately interpret these results. Subsequent application of molecular biotechnology to interest candidate genes can facilitate the breeding production of alkali salt resistance in rapeseed. For example, gene editing technology can be used to knock out alkaline salt-sensitive genes in sensitive materials to enhance their resistance. Genes positively regulating alkaline salt resistance can be overexpressed in resistant cultivars to further enhance their resistance.

## 5. Conclusions

In summary, alkaline salts can affect membrane permeability by stimulating many strong physiological responses to alkaline salts, and the damage to mesophyll cells in tolerant canola is attenuated and delayed. The responses include enhanced osmoregulation through the addition of soluble protein and proline, and more favorable ROS scavenging through higher SOD and POD activities. RNA-seq analysis demonstrated that high expression genes in lines 2205 and 1423 participated in different metabolic pathways. In line 2205, high-expression genes participated in positive pathways such as carbohydrate metabolism, photosynthetic processes, and ROS regulating, which enhanced its tolerance. Twelve candidate genes were identified in multiple target pathways which might be the key genes responsible for alkaline salt responses in rapeseed.

## Data Availability

The Global Map of Salt-affected Soils (GSASmap) V1.0.0 was released by the Status of the World’s Soil Resources, Food and Agriculture Organization of the United Nations (https://www.fao.org/soils-portal/data-hub/soil-maps-and-databases/global-map-of-salt-affected-soils/en/, accessed on 18 April 2022). RNA-seq analyses were performed using BMKCloud (www.biocloud.net). The reference genome of *B. napus* was downloaded from http://www.genoscope.cns.fr/brassicanapus/data/ (accessed on 20 September 2021). The RNA-seq data have been submitted to NCBI BioProject with BioProject ID: PRJNA682529. The datasets supporting the conclusions of this article are included within the article and its additional files.

## Supplementary Materials

The following supporting information can be downloaded at: https://www.mdpi.com/article/10.3390/genes13081493/s1. Table S1: Primes for qRT-PCR verification; Table S2: The sequencing results of 18 RNA libraries; Table S3: Differentially expressed genes on different groups; Table S4: The differentially expressed genes for further functional analysis; Table S5: Target pathways and annotated DEGs; Table S6: Fifty-five DEGs overlapped on multiple pathways.

## References

[1] van Zelm E., Zhang Y., Testerink C. (2020). Salt Tolerance Mechanisms of Plants. Annu. Rev. Plant Biol..

[2] Annunziata M.G., Ciarmiello L.F., Woodrow P., Maximova E., Fuggi A., Carillo P. (2016). Durum Wheat Roots Adapt to Salinity Remodeling the Cellular Content of Nitrogen Metabolites and Sucrose. Front. Plant Sci..

[3] Yang J. (2008). Development and Prospect of the Research on Salt-Affected Soils in China. Acta Pedol. Snica.

[4] Yang J., Yao R., Wang X., Xie W., Zhang X., Zhu W., Zhang L., Sun R. (2022). Research on Salt-Affected Soils in China: History, Status Quo and Prospect. Acta Pedol. Snica.

[5] Tuyen D.D., Lal S.K., Xu D.H. (2010). Identification of a Major Qtl Allele from Wild Soybean (*Glycine Soja* Sieb. & Zucc.) for Increasing Alkaline Salt Tolerance in Soybean. Theor. Appl. Genet..

[6] Munns R., Tester M. (2008). Mechanisms of Salinity Tolerance. Annu. Rev. Plant Biol..

[7] Li N., Zhang Z., Gao S., Lv Y., Chen Z., Cao B., Xu K. (2021). Different Responses of Two Chinese Cabbage (*Brassica rapa* L. ssp. *pekinensis*) Cultivars in Photosynthetic Characteristics and Chloroplast Ultrastructure to Salt and Alkali Stress. Planta.

[8] Ruili L.I., Shi F., Fukuda K., Yang Y. (2010). Effects of Salt and Alkali Stresses on Germination, Growth, Photosynthesis and Ion Accumulation in Alfalfa *(Medicago Sativa* L.). Soil Sci. Plant Nutr..

[9] Choi W.G., Toyota M., Kim S.H., Hilleary R., Gilroy S. (2014). Salt Stress-Induced Ca^2+^ Waves Are Associated with Rapid, Long-Distance Root-to-Shoot Signaling in Plants. Proc. Natl. Acad. Sci. USA.

[10] Geng Y., Wu R., Wee C.W., Xie F., Wei X., Chan P.M., Tham C., Duan L., Dinneny J.R. (2013). A Spatio-Temporal Understanding of Growth Regulation During the Salt Stress Response in *Arabidopsis*. Plant Cell.

[11] Sun J., Dai S., Wang R., Chen S., Li N., Zhou X., Lu C., Shen X., Zheng X., Hu Z. (2009). Calcium Mediates Root K+/Na+ Homeostasis in Poplar Species Differing in Salt Tolerance. Tree Physiol..

[12] Almeida D.M., Oliveira M.M., Saibo N.J.M. (2017). Regulation of Na^+^ and K^+^ Homeostasis in Plants: Towards Improved Salt Stress Tolerance in Crop Plants. Genet. Mol. Biol..

[13] Miller G., Suzuki N., Ciftci-Yilmaz S., Mittler R. (2010). Reactive Oxygen Species Homeostasis and Signalling during Drought and Salinity Stresses. Plant Cell Environ..

[14] Bose J., Munns R., Shabala S., Gilliham M., Pogson B., Tyerman S.D. (2017). Chloroplast Function and Ion Regulation in Plants Growing on Saline Soils: Lessons from Halophytes. J. Exp. Bot..

[15] Dong S., Zhang J., Beckles D.M. (2018). A Pivotal Role for Starch in the Reconfiguration of ^14^c-Partitioning and Allocation in *Arabidopsis thaliana* under Short-Term Abiotic Stress. Sci. Rep..

[16] Li X., Li S., Wang J., Lin J. (2020). Exogenous Abscisic Acid Alleviates Harmful Effect of Salt and Alkali Stresses on Wheat Seedlings. Int. J. Environ. Res. Public Health.

[17] Guo R., Yang Z., Li F., Yan C., Zhong X., Liu Q., Xia X., Li H., Zhao L. (2015). Comparative Metabolic Responses and Adaptive Strategies of Wheat (*Triticum Aestivum*) to Salt and Alkali Stress. BMC Plant Biol..

[18] Gong B., Wen D., Bloszies S., Li X., Wei M., Yang F.J., Shi Q.H., Wang X.F. (2014). Comparative Effects of Nacl and Nahco_3_ Stresses on Respiratory Metabolism, Antioxidant System, Nutritional Status, and Organic Acid Metabolism in Tomato Roots. Acta Physiol. Plant.

[19] Li X., Zheng H., Wu W., Liu H., Wang J., Jia Y., Li J., Yang L., Lei L., Zou D. (2020). Qtl Mapping and Candidate Gene Analysis for Alkali Tolerance in *Japonica* Rice at the Bud Stage Based on Linkage Mapping and Genome-Wide Association Study. Rice.

[20] Li N., Zheng H., Cui J., Wang J., Liu H., Sun J., Liu T., Zhao H., Lai Y., Zou D. (2019). Genome-Wide Association Study and Candidate Gene Analysis of Alkalinity Tolerance in *Japonica* Rice Germplasm at the Seedling Stage. Rice.

[21] He Y., Dong Y., Yang X., Guo D., Qian X., Yan F., Wang Y., Li J., Wang Q. (2020). Functional Activation of a Novel R2r3-Myb Protein Gene, Gmmyb68, Confers Salt-Alkali Resistance in Soybean (*Glycine Max* L.). Genome.

[22] He Y., Yang X., Xu C., Guo D., Niu L., Wang Y., Li J., Yan F., Wang Q. (2018). Overexpression of a Novel Transcriptional Repressor *Gmmyb3a* Negatively Regulates Salt-Alkali Tolerance and Stress-Related Genes in Soybean. Biochem. Biophys. Res. Commun..

[23] Zhu D., Cai H., Luo X., Bai X., Deyholos M.K., Chen Q., Chen C., Ji W., Zhu Y. (2012). Over-Expression of a Novel Jaz Family Gene from *Glycine Soja*, Increases Salt and Alkali Stress Tolerance. Biochem. Biophys. Res. Commun..

[24] Guan Q.J., Wang L.F., Bu Q.Y., Wang Z.Y. (2014). The Rice Gene *Oszfp6* Functions in Multiple Stress Tolerance Responses in Yeast and *Arabidopsis*. Plant Physiol. Biochem..

[25] Guan Q.J., Ma H.Y., Wang Z.J., Wang Z.Y., Bu Q.Y., Liu S.K. (2016). A Rice Lsd1-Like-Type Zfp Gene *Oslol5* Enhances Saline-Alkaline Tolerance in Transgenic *Arabidopsis thaliana*, Yeast and Rice. BMC Genom..

[26] An Y., Yang X.X., Zhang L., Zhang J., Du B., Yao L., Li X.T., Guo C. (2020). Alfalfa *Mscbl4* Enhances Calcium Metabolism but Not Sodium Transport in Transgenic Tobacco under Salt and Saline-Alkali Stress. Plant Cell Rep..

[27] Du B., Chen N., Song L., Wang D., Cai H., Yao L., Li X., Guo C. (2021). Alfalfa (*Medicago sativa* L.) *Mscml46* Gene Encoding Calmodulin-Like Protein Confers Tolerance to Abiotic Stress in Tobacco. Plant Cell Rep..

[28] Zheng M., Zhang L., Tang M., Liu J., Liu H., Yang H., Fan S., Terzaghi W., Wang H., Hua W. (2020). Knockout of Two *Bnamax1* Homologs by Crispr/Cas9-Targeted Mutagenesis Improves Plant Architecture and Increases Yield in Rapeseed (*Brassica napus* L.). Plant Biotechnol. J..

[29] Mohamed I.A.A., Shalby N., El-Badri A.M., Batool M., Wang C., Wang Z., Salah A., Rady M.M., Jie K., Wang B. (2022). Rna-Seq Analysis Revealed Key Genes Associated with Salt Tolerance in Rapeseed Germination through Carbohydrate Metabolism, Hormone, and Mapk Signaling Pathways. Ind. Crops Prod..

[30] Zhang F., Zhu G., Du L., Shang X., Cheng C., Yang B., Hu Y., Cai C., Guo W. (2016). Genetic Regulation of Salt Stress Tolerance Revealed by Rna-Seq in Cotton Diploid Wild Species, *Gossypium davidsonii*. Sci. Rep..

[31] Hoagland D.R., Arnon D.S. (1950). The Water Culture Method for Growing Plants without Soil. Calif. Agric. Exp. Stn. Circ..

[32] Lutts S., Kinet J.M., Bouharmont J. (1996). Nacl-Induced Senescence in Leaves of Rice ( *Oryza Sativa* L.) Cultivars Differing in Salinity Resistance. Ann. Bot..

[33] Zhao L., Liu F., Xu W., Di C., Zhou S., Xue Y., Yu J., Su Z. (2009). Increased Expression of *Osspx1* Enhances Cold/Subfreezing Tolerance in Tobacco and *Arabidopsis thaliana*. Plant Biotechnol. J..

[34] Kannangara C.G., Woolhouse H.W. (1968). Changes in the Enzyme Activity of Soluble Protein Fractions in the Course of Foliar Senescence in *Perilla frutescens* (L.) Britt. New Phytol..

[35] Beauchamp C., Fridovich I. (1971). Superoxide Dismutase: Improved Assays and an Assay Applicable to Acrylamide Gels. Anal. Biochem..

[36] Zhao X., Wei P., Liu Z., Yu B., Shi H. (2016). Soybean Na^+^/H^+^ Antiporter *Gmssos1* Enhances Antioxidant Enzyme Activity and Reduces Na^+^ Accumulation in *Arabidopsis* and Yeast Cells under Salt Stress. Acta Physiol. Plant..

[37] Kim D., Langmead B., Salzberg S.L. (2015). Hisat: A Fast Spliced Aligner with Low Memory Requirements. Nat. Methods.

[38] Florea L., Song L., Salzberg S.L. (2013). Thousands of Exon Skipping Events Differentiate among Splicing Patterns in Sixteen Human Tissues. F1000Research.

[39] Anders S., Huber W. (2010). Differential Expression Analysis for Sequence Count Data. Genome Biol.

[40] Young M.D., Wakefield M.J., Smyth G.K., Oshlack A. (2010). Gene Ontology Analysis for Rna-Seq: Accounting for Selection Bias. Genome Biol..

[41] Xie C., Mao X., Huang J., Ding Y., Wu J., Dong S., Kong L., Gao G., Li C.Y., Wei L. (2011). Kobas 2.0: A Web Server for Annotation and Identification of Enriched Pathways and Diseases. Nucleic Acids Res..

[42] Livak K.J., Schmittgen T.D. (2001). Analysis of Relative Gene Expression Data Using Real-Time Quantitative Pcr and the 2^−ΔΔCT^ Method. Methods.

[43] Tsai Y.C., Chen K.C., Cheng T.S., Lee C., Lin S.H., Tung C.W. (2019). Chlorophyll Fluorescence Analysis in Diverse Rice Varieties Reveals the Positive Correlation between the Seedlings Salt Tolerance and Photosynthetic Efficiency. BMC Plant Biol..

[44] Watson S.J., Sowden R.G., Jarvis P. (2018). Abiotic Stress-Induced Chloroplast Proteome Remodelling: A Mechanistic Overview. J. Exp. Bot..

[45] Lang L., Xu A., Ding J., Zhang Y., Zhao N., Tian Z., Liu Y., Wang Y., Liu X., Liang F. (2017). Quantitative Trait Locus Mapping of Salt Tolerance and Identification of Salt-Tolerant Genes in *Brassica napus* L.. Front. Plant Sci..

[46] Ibrahim E.B., Mohamed S., Stephen B., Harold B., Sabah M. (2017). Cell Membrane Stability and Association Mapping for Drought and Heat Tolerance in a Worldwide Wheat Collection. Sustainability.

[47] Thalmann M., Santelia D. (2017). Starch as a Determinant of Plant Fitness under Abiotic Stress. New Phytol..

[48] Mohamed I.A.A., Shalby N., Bai C., Qin M., Agami R.A., Jie K., Wang B., Zhou G. (2020). Stomatal and Photosynthetic Traits Are Associated with Investigating Sodium Chloride Tolerance of *Brassica napus* L. Cultivars. Plants.

[49] Ghosh U.K., Islam M.N., Siddiqui M.N., Cao X., Khan M.A.R. (2022). Proline, a Multifaceted Signalling Molecule in Plant Responses to Abiotic Stress: Understanding the Physiological Mechanisms. Plant Biol..

[50] Hasanuzzaman M., Bhuyan M., Zulfiqar F., Raza A., Mohsin S.M., Mahmud J.A., Fujita M., Fotopoulos V. (2020). Reactive Oxygen Species and Antioxidant Defense in Plants under Abiotic Stress: Revisiting the Crucial Role of a Universal Defense Regulator. Antioxidants.

[51] Parida A.K., Das A.B. (2005). Salt Tolerance and Salinity Effects on Plants: A Review. Ecotoxicol. Environ. Saf..

[52] Krzesłowska M. (2011). The Cell Wall in Plant Cell Response to Trace Metals: Polysaccharide Remodeling and Its Role in Defense Strategy. Acta Physiol. Plant..

[53] Li Y., Guo Q., Liu P., Huang J., Zhang S., Yang G., Wu C., Zheng C., Yan K. (2021). Dual Roles of the Serine/Arginine-Rich Splicing Factor Sr45a in Promoting and Interacting with Nuclear Cap-Binding Complex to Modulate the Salt-Stress Response in *Arabidopsis*. New Phytol..

[54] Hu Q., Chen Y., Zhao Y., Gu J., Ma M., Li H., Li C., Wang Z.Y. (2021). Overexpression of *Scl30a* from Cassava (*Manihot Esculenta*) Negatively Regulates Salt Tolerance in *Arabidopsis*. Funct. Plant Biol..

[55] Wang W., Pang J., Zhang F., Sun L., Yang L., Zhao Y., Yang Y., Wang Y., Siddique K.H.M. (2021). Integrated Transcriptomics and Metabolomics Analysis to Characterize Alkali Stress Responses in Canola (*Brassica napus* L.). Plant Physiol. Biochem..

[56] Abd El-Moneim D., Contreras R., Silva-Navas J., Gallego F.J., Figueiras A.M., Benito C. (2015). On the Consequences of Aluminium Stress in Rye: Repression of Two Mitochondrial Malate Dehydrogenase Mrnas. Plant Biol..

[57] Kim S.C., Guo L., Wang X. (2020). Nuclear Moonlighting of Cytosolic Glyceraldehyde-3-Phosphate Dehydrogenase Regulates Arabidopsis Response to Heat Stress. Nat. Commun..

[58] Liu T., Fang H., Liu J., Reid S., Hou J., Zhou T., Tian Z., Song B., Xie C. (2017). Cytosolic Glyceraldehyde-3-Phosphate Dehydrogenases Play Crucial Roles in Controlling Cold-Induced Sweetening and Apical Dominance of Potato (*Solanum tuberosum* L.) Tubers. Plant Cell Environ..

[59] Schneider M., Knuesting J., Birkholz O., Heinisch J.J., Scheibe R. (2018). Cytosolic Gapdh as a Redox-Dependent Regulator of Energy Metabolism. BMC Plant Biol..

[60] Vescovi M., Zaffagnini M., Festa M., Trost P., Lo Schiavo F., Costa A. (2013). Nuclear Accumulation of Cytosolic Glyceraldehyde-3-Phosphate Dehydrogenase in Cadmium-Stressed Arabidopsis Roots. Plant Physiol..

[61] Nie X., Singh R.P., Tai G.C. (2002). Molecular Characterization and Expression Analysis of 1-Aminocyclopropane-1-Carboxylate Oxidase Homologs from Potato under Abiotic and Biotic Stresses. Genome.

[62] Kachroo A., He Z., Patkar R., Zhu Q., Zhong J., Li D., Ronald P., Lamb C., Chattoo B.B. (2003). Induction of H_2_O_2_ in Transgenic Rice Leads to Cell Death and Enhanced Resistance to Both Bacterial and Fungal Pathogens. Transgenic Res..

[63] Wu G., Shortt B.J., Lawrence E.B., Levine E.B., Fitzsimmons K.C., Shah D.M. (1995). Disease Resistance Conferred by Expression of a Gene Encoding H_2_O_2_-Generating Glucose Oxidase in Transgenic Potato Plants. Plant Cell.

[64] Igarashi D., Miwa T., Seki M., Kobayashi M., Kato T., Tabata S., Shinozaki K., Ohsumi C. (2003). Identification of Photorespiratory *Glutamate:Glyoxylate Aminotransferase* (*Ggat*) Gene in *Arabidopsis*. Plant J..

[65] Yang H., Deng L., Liu H., Fan S., Hua W., Liu J. (2019). Overexpression of *Bnaaox1b* Confers Tolerance to Osmotic and Salt Stress in Rapeseed. G3.

[66] Zhang H., Yang B., Liu W.Z., Li H., Wang L., Wang B., Deng M., Liang W., Deyholos M.K., Jiang Y.Q. (2014). Identification and Characterization of Cbl and Cipk Gene Families in Canola (*Brassica napus* L.). BMC Plant Biol..

